# Effect of feeding *Tithonia diversifolia* zinc oxide nanoparticle emulsion on glutathione peroxidase and anti-insulin production in diabetic nephropathy Wistar rats

**DOI:** 10.14202/vetworld.2025.397-407

**Published:** 2025-02-19

**Authors:** Iwan Sahrial Hamid, Lailatul Muniroh, Salipudin Tasil Maslamama, Rondius Solfaine

**Affiliations:** 1 Department of Veterinary Science, Faculty of Veterinary Medicine, Universitas Airlangga, Surabaya, 60115, Indonesia; 2 Department of Nutrition, Faculty of Public Health, Universitas Airlangga, Kampus C UNAIR, Mulyorejo, Surabaya, 60115, Indonesia; 3 Department of Agricultural Biotechnology, Faculty of Agriculture, Eskişehir Osmangazi Üniversitesi, Eskişehir, 26040, Turkey; 4 Laboratory of Pathology, Faculty of Veterinary Medicine, Universitas Wijaya Kusuma Surabaya, Surabaya, 60225, Indonesia

**Keywords:** anti-insulin, diabetic nephropathy, healthy lifestyle, inflammation, oxidative stress, *Tithonia diversifolia*, zinc oxide nanoparticles

## Abstract

**Background and Aim:**

Diabetic nephropathy (DN) is a major complication of diabetes mellitus characterized by oxidative stress and inflammation. Conventional treatments often fail to prevent its progression. This study investigates the therapeutic potential of *Tithonia diversifolia* zinc oxide nanoparticle emulsion (TDNP) in mitigating DN by enhancing antioxidant and immunomodulatory mechanisms. This study aimed to evaluate the effect of TDNP on oxidative stress markers, inflammation, and insulin activity in streptozotocin (STZ)-induced DN rats.

**Materials and Methods:**

Male Wistar rats (n = 24) were divided into four groups: control (saline), positive control (0.1% zinc oxide suspension), treatment (TDNP at 100 mg/kg body weight), and comparison (quercetin at 5 mg/kg body weight). DN was induced using STZ and nicotinamide. Blood glucose, creatinine, urea, gamma-glutamyl transferase (γ-GT), and high-density lipoprotein cholesterol levels were assessed. Oxidative stress markers (superoxide dismutase [SOD], glutathione peroxidase [GPx]), inflammatory cytokines (TNF-α), and immunohistochemical indicators (anti-insulin, interferon-gamma [IFN-γ]) were measured. Data were analyzed using a one-way analysis of variance and Kruskal–Wallis tests.

**Results:**

TDNP treatment significantly reduced blood glucose, creatinine, urea, γ-GT, and TNF-α levels (p ≤ 0.05), while increasing SOD, GPx, and anti-insulin levels compared with the positive control. Histopathological analysis showed decreased necrosis and inflammation in pancreatic and renal tissues. Immunohistochemistry revealed enhanced anti-insulin and reduced IFN-γ expression in TDNP-treated rats, indicating improved immune regulation and oxidative stress mitigation.

**Conclusion:**

TDNP demonstrates potent antioxidative and anti-inflammatory effects, effectively improving glucose metabolism and kidney function in DN. These findings highlight TDNP as a promising therapeutic agent for managing DN.

## INTRODUCTION

Diabetes mellitus is characterized by elevated blood sugar levels resulting from impaired insulin secretion, action, or resistance. This condition leads to organ damage and various complications in the host. Diabetes mellitus is a major cause of progressive kidney failure that often necessitates dialysis or transplantation. One of the emerging types of diabetes, diabetic nephropathy (DN), which is prevalent among patients with diabetes and the leading cause of end-stage renal failure globally, is marked by structural and functional abnormalities in the kidneys. Poor glycemic control and the accumulation of advanced glycation end-products are critical factors in its development [1].

One pivotal aspect of chronic hyperglycemia is the occurrence of complications such as DN, neuropathy, and retinopathy. DN, which can progress to end-stage renal disease, involves mechanisms such as aldose reductase and protein kinase C activation and the accumulation of advanced glycation end products, all of which contribute to nephron damage. The condition is marked by partial kidney function loss, nephrotic syndrome, glomerulosclerosis, albuminuria, low glomerular filtration rate, high blood pressure, and impaired fluid excretion. The pathogenesis includes reduced nitric oxide synthase and increased inflammatory mediators. The key risk factors are hyperglycemia, hyperlipidemia, and hypertension [2, 3].

The pathogenesis of diabetes is influenced by factors such as obesity, insulin receptor inhibition, and elevated blood sugar and fat levels. Type 2 diabetes is associated with gene polymorphisms and lifestyle factors. A mouse model containing a high-fat diet and low-dose streptozotocin (STZ) effectively mimicked the progression of diabetes, facilitating the evaluation of potential therapies [4–7].

STZ, often combined with nicotinamide, is used in research to damage pancreatic beta cells and stop insulin secretion in animals. STZ induces the generation of superoxide free radicals, which are then converted to highly reactive hydroxyl radicals through the Fenton reaction. This process leads to the rapid destruction of beta cells by increasing cytosolic calcium levels. Nicotinamide provides partial protection to beta cells, resulting in a model closely resembling type 2 diabetes, making it suitable for studying potential therapies. Nicotinamide helps partially protect beta cells, creating a model that closely mimics type 2 diabetes for studying potential therapies [8, 9].

The moonflower plant (*Tithonia diversifolia*) is traditionally used to treat high blood sugar, diarrhea, malaria, hematoma, liver inflammation, tumors, and wounds. It contains active compounds such as terpenoids and flavonoids. Research indicates that the plant possesses anti-inflammatory, anti-tumor, and anti-diabetic properties and exerts a protective effect on kidney tubules in experimental animal models [10, 11].

DN poses a major challenge in diabetes management, often requiring dialysis or transplantation. Current therapies focus on hyperglycemia, hypertension, and dyslipidemia but are insufficient to prevent disease progression in all cases. Inflammatory mediators, oxidative stress, and endothelial dysfunction are key contributors. *T. diversifolia*, rich in flavonoids and terpenoids, has the potential to manage diabetes. Combining it with zinc oxide nanoparticles (ZnO-NPs) may enhance its bioavailability and therapeutic effects, offering a promising approach to mitigate DN.

This study aimed to investigate the impact of zinc-oxide nanoparticle *T. diversifolia* emulsion (TDNP) on increasing glutathione peroxidase (GPx) and anti-insulin (anti-ins) levels in DN rats.

## MATERIALS AND METHODS

### Ethical approval

The study was approved by the Animal Care and Use Committee of the Faculty of Veterinary, Universitas Airlangga, Surabaya, Indonesia (No. 1.KEH.097.07.2024).

### Study period and location

The study was conducted from June 2024 to August 2024 at the Institute of Tropical Disease, Universitas Airlangga, and the Animal Research Laboratory, Universitas Wijaya Kusuma Surabaya, Indonesia.

### Nanoparticle emulsion and plant extraction

*T. diversifolia* was obtained from rural areas in Magelang, Central Java, Indonesia. The plant was identified by Laboratorium Penelitian dan Pengujian Terpadu (LPPT), Universitas Gadjah Mada (UGM). The leaves of *T. diversifolia* were extracted using 70% ethanol through maceration for 6–24 h, following the method outlined by Muniroh *et al*. [12]. Subsequently, zinc oxide (ZnO) nanoparticles were synthesized by dissolving 1.5 g of zinc sulfate heptahydrate in 162.5 mL of distilled water and adding 2 g of sodium hydroxide in 50 mL of deionized water dropwise with magnetic stirring for 30 min. The precipitates were filtered, washed with pure water, dried at 60°C for 24 h, and calcined at 400°C for 2 h. For the emulsion, 7.5 mL of virgin coconut oil, 52.5-mL Tween 80, and 25 mL of polyethylene glycol were heated to 70°C. The aqueous phase was gradually added to the oil phase while stirring continuously until saponification occurred. ZnO nanocrystals were added at a concentration of 1%. *T. diversifolia* zinc-oxide nanoparticle (TDNP) emulsion was prepared by mixing a *T. diversifolia* extract solution with a ZnO solution in a 9:1 ratio, resulting in a 1 mM concentration. The mixture was then agitated at 28°C for several hours [13].

### Nanoparticle analysis

The particle size analyzer (PSA) instrument was used to analyze the size and size distribution of ZnO nanoparticles obtained from *T. diversifolia* (BK-802N, Biobase, Shandong, China). The ZnO nanoparticle sample was dissolved in ethanol at a concentration of 10 ppm to form an emulsion. After homogenization, the emulsion was placed in a cuvette and analyzed using a PSA instrument. Size distribution analysis was complemented by graphic images obtained through scanning electron microscopy (SEM) using equipment (Thermo Fisher Scientific, Massachusetts, USA, Cat No: 4491809006). SEM provided visual insights into size variations and the structural characteristics and morphology of the investigated ZnO nanoparticles [14].

### Biological experiment

Male Wistar rats aged 2–3 months and weighing 200–250 g (*Rattus norvegicus*) were obtained from the Animal Laboratory, Faculty of Medicine at Universitas Airlangga for this study. Male Wistar rats were used to avoid hormonal fluctuations in females that could affect metabolic and renal parameters, ensuring consistent results. After a 7-day acclimatization period in laboratory conditions with standard food pellets and tap water at 25°C–27°C, rats were divided into four groups, each consisting of six animals. The control group (S0) received normal saline, the positive group (S1) received a 0.1% ZnO suspension, the treatment group (S2) received TDNP orally at a dosage of 100 mg/kg body weight, and the comparison group (S3) received quercetin at 5 mg/kg body weight for 7 days. Rats in groups S1, S2, and S3 received a single intraperitoneal dose of STZ (Sigma-Aldrich USA) at 100 mg/kg body weight or nicotinamide (Sigma-Aldrich) at a dose of 200 mg/kg body weight orally for 1 week. On the 8^th^ day, the rats were sacrificed, and blood and tissue samples were collected. The kidney and pancreas specimens were preserved in 10% buffered neutral formalin for subsequent immunohistochemical and hematoxylin-eosin staining.

### Biochemical evaluation

The concentrations of blood glucose (BG), creatinine, urea, gamma-glutamyl transferase (γ-GT), and high-density lipoprotein cholesterol (HDL-C) were measured using the colorimetric method (Robert Reil, Germany). In this procedure, 10 µL of serum was mixed with 1 mL of the appropriate reagent and incubated for 10 min. The absorbance of the resulting colored solution was then measured at 546 nm. BG and serum cholesterol concentrations were determined by comparing the absorbance values of the samples with those of standard solutions [15]. The concentrations of tumor necrosis factor-alpha (TNF-α), superoxide dismutase (SOD), and GPx were measured using the quantitative sandwich enzyme-linked immunosorbent assay technique (Elabscience, Wuhan, China). A microplate precoated with rat monoclonal antibodies for TNF-α, SOD, and GPx was used. Samples were added to the microplate, followed by incubation and washing. An enzyme-linked detection antibody for leptin and adiponectin was then introduced, and a substrate for the enzyme was added, resulting in a colorimetric reaction measured at 450 nm [16].

### Histopathological study

Kidney interferon-gamma (IFN-γ) and pancreatic anti-ins concentrations were determined using indirect immune peroxidase monoclonal antibodies and secondary anti-peroxidase antibodies. These antibodies were stained with diaminobenzidine (Bioss, USA), labeled with a streptavidin-biotin complex, and analyzed using the Starr Trek Universal detection system (ScyTek, USA) [17].

H&E scores: 0 for normal tissue, 1 for 1%–25% degeneration/infiltration, 3 for 26%–50%, 5 for 51%–75%, and 7 for 76%–100% across multiple fields. Immunohistochemistry scores: 0 for no immunoreactivity, 3 for 1%–25% with mild intensity, 5 for 26%–50% with moderate intensity, 7 for 51%–75% with moderate intensity, and 9 for 76%–100% with strong intensity across multiple fields at 100× magnification [18, 19].

### Statistical analysis

Statistical analyses were conducted using the Statistical Package for the Social Sciences 25.0 (IBM Corp., USA), with significance set at p ≤ 0.05. Data are presented as mean ± standard deviation for parametric and median (interquartile range) for non-parametric data. Normality was assessed through the Shapiro–Wilk test and homogeneity through Levene’s test. One-way analysis of variance with Duncan’s *post hoc* test was used for normally distributed data, while the Kruskal–Wallis test with Mann–Whitney U (Bonferroni correction) was applied for non-parametric data. Pearson’s or Spearman’s correlation analyzed biochemical markers, and the Chi-square or Fisher’s exact test assessed categorical data.

## RESULTS

**Figure 1:**
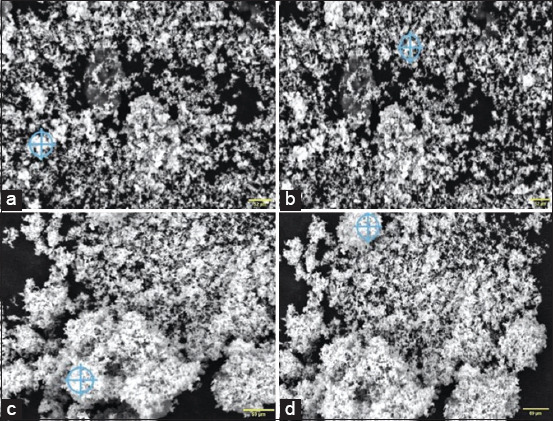
(a–d) The morphology of the *Tithonia diversifolia* zinc oxide nanoparticle emulsion was investigated using scanning electron microscopy, revealing particle sizes predominantly within the range of 14.01 nm or less.

Analysis of *T. diversifolia* ZnO nanoparticle (TDNP) emulsion using a PSA and SEM confirmed the nanoparticle characteristics of TDNP. The criteria include an average diameter of all detected particle sizes with a dispersion index of 0.69, predominantly within a size range of less than or equal to 14.01 nm. Specifically, particles measuring 10% of the total particle volume fall within this size range, while particles smaller than 35.6 nm constitute 50% of the total particles, and particles smaller than 90.2 nm make up 90% of the total particle volume (Figure 1).

The typical symptoms of DN were high BG and urea, gamma-GT, HDL-C, and creatinine concentrations resulting from insulin resistance, impaired insulin secretion, and acute kidney failure. Hence, a standard assessment for evaluating the metabolic status of individuals or animals with DN includes measuring BG, creatinine, urea, and insulin concentrations.

Significantly reduced concentrations of BG, creatinine, urea, γ-GT, and HDL-C were observed in the S2 and S3 groups (p ≤ 0.05) compared with the S1 group. In the S2 and S3 groups, the concentrations of SOD and GPx were significantly increased (p ≤ 0.05) compared with the S1 group. The S2 group showed the lowest average BG concentration, with significant differences (p ≤ 0.05) observed among all groups and between groups S0 and S1, S1 and S2, as well as S1 and S3. The S2 group exhibited the highest average concentrations of SOD and GPx, reflecting enhanced antioxidant activity compared with the other groups. Conversely, the S1 group had the highest average TNF-α concentration, indicating a heightened inflammatory response. Significant differences (p ≤ 0.05) in insulin and TNF-α concentrations were observed between groups S0 and S1, S1 and S2, and S1 and S3. Similarly, significant differences in SOD and GPx levels were observed between S0 and S1, S1 and S2, and S1 and S3. However, no significant differences in GPx concentrations were noted between S2 and S3, as shown in Table 1. This suggests that treatment with both TDNP and quercetin effectively enhances GPx levels comparably.

**Table 1:** Concentration of BG, creatinine, urea, γ-GT, HDL-C, TNF-α, SOD, GPx, and insulin in various groups.

Group	BG (mg/dL)	Creatinine (mg/dL)	Urea (mg/dL)	γ-GT (IU/L)	HDL-C (mg/dL)	TNF-α (pg/mL)	SOD (mg/dL)	GPx (pg/mL)	Insulin (mg/dL)
S0 (n = 6)	87.00^a^ ± 5.76	0.64^a^ ± 0.04	82.03^a^ ± 17.88	4.17^a^ ± 1.16	59.83^a^ ± 1.85	41.81^a^ ± 3.93	1.40^a^ ± 0.35	181.38^a^ ± 16.55	0.14^a^ ± 0.03
S1 (n = 6)	267.67^b^ ± 24.28	0.92^b^ ± 0.06	187.23^b^ ± 19.22	10.33^b^ ± 1.72	56.67^b^ ± 1.53	99.92^b^ ± 9.59	1.80^b^ ± 0.45	146.60^b^ ± 19.87	0.04^b^ ± 0.01
S2 (n = 6)	132.17^c^ ± 43.54	0.65^a^ ± 0.03	110.23^c^ ± 33.39	4.17^a^ ± 1.16	48.33^c^ ± 1.03	47.14^c^ ± 3.33	2.0^c^ ± 0.49	177.49^c^ ± 13.06	0.15^c^ ± 0.05
S3 (n = 6)	142.83^d^ ± 31.30	0.63^a^ ± 0.07	111.45^c^ ± 58.16	6.33^c^ ± 3.44	69.17^d^ ± 1.52	64.30^d^ ± 3.43	1.70^d^ ± 0.26	176.66^c^ ± 16.88	0.07^d^ ± 0.01

Values are presented as mean ± standard deviation. ^a,b,c,d^Different superscripts of similar column show significant differences (p < 0.05). S0=Control group, S1=Positive control group, S2=Induction + TDNP group, S3=Induction+quercetin group, γ-GT=Gamma-glutamyl transferase, HDL-C=High-density lipoprotein cholesterol, TNF-α=Tumor necrosis factor-alpha, SOD=Superoxide dismutase, GPx=Glutathione peroxidase, BG=Blood glucose

Histopathology scores for necrotic and infiltrated cells were significantly higher in group S1 than in group S2 (p ≤ 0.05). Moreover, immune positive scores for anti-Ins and IFN-γ expressions significantly differed between the treatment group (S2) and S1 groups (p ≤ 0.05) (Table 2).

**Table 2:** Comparison of different histopathological and immunohistochemistry scores among the groups.

Group	Pancreas (HE)		Kidney (IHC)	Pancreas (IHC)
		
	Necrotic cell	Infiltration cell	IFN-γ	Anti-ins
S0 (n = 6)	1.50^a^ ± 0.55	1.20^a^ ± 0.41	0.25^a^ ± 0.50	2.00^a^ ± 0.74
S1 (n = 6)	2.00^b^ ± 0.74	1.75^b^ ± 0.90	9.50^b^ ± 1.73	1.50^b^ ± 0.55
S2 (n =6)	2.00^b^ ± 0.74	1.80^b^ ± 0.86	3.50^c^ ± 1.73	2.00^a^ ± 0.74
S3 (n = 6)	1.50^a^ ± 0.54	1.20^a^ ± 0.41	4.25^c^ ± 1.26	1.50^b^ ± 0.54

The superscript in the similar column indicates significant differences (p ≤ 0.05). HE=Hematoxylin-eosin, IHC=Immunohistochemistry, IFN-γ=Interferon-gamma, anti-ins=Anti-insulin, S0=Control group, S1=Positive control group, S2=Induction + TDN group, S3=Induction + quercetin group

**Figure 2:**
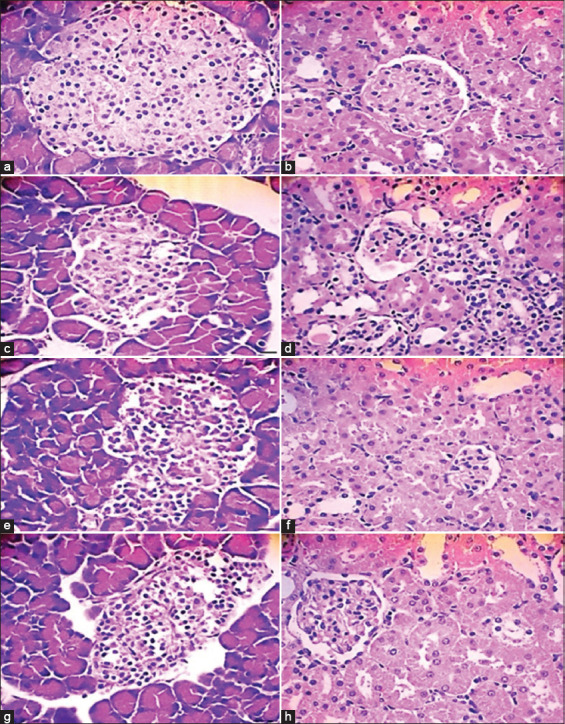
Histomorphological examination of the islets of Langerhans and kidneys in control and treatment groups, showing morphological changes such as hydropic degeneration, necrosis, and mononuclear cell infiltration. (a) Control rats in the islets of Langerhans, (b) Control rats in the kidneys, (c) STZ-Nam-induced rats in the islets of Langerhans, (d) STZ-Nam-induced rats in the kidneys, (e) STZ-Nam-induced rats treated with TDN extract in the islets of Langerhans, (f) STZ-Nam-induced rats treated with TDN extract in the kidneys, (g) STZ-Nam-induced rats treated with quercetin in the islets of Langerhans, and (h) STZ-Nam-induced rats treated with quercetin in the kidneys. Samples were stained with hematoxylin and eosin (H&E) and observed at 100× magnification.

**Figure 3:**
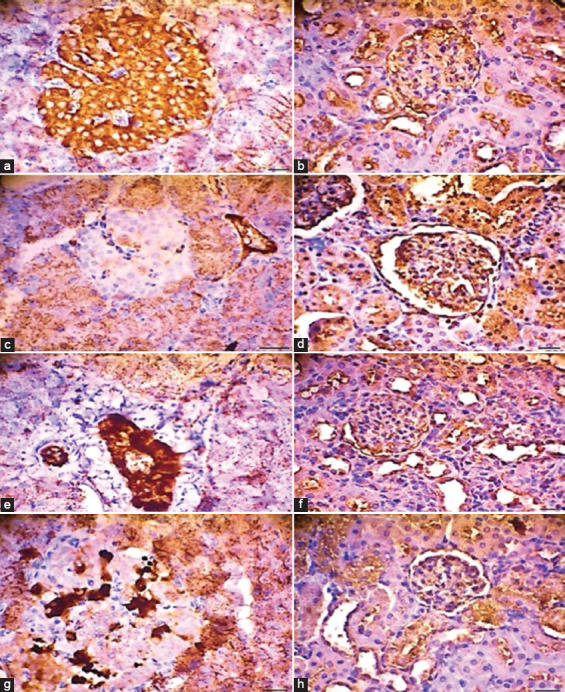
Histomorphological examination of monoclonal antibodies against anti-Insulin (anti-Ins) positive cells and renal interferon-gamma positivity in control and treatment groups. (a) Control rats showing anti-Ins-positive cells in the beta-cell area of the pancreas, indicating insulin secretion, (b) Control rats showing renal interferon-gamma positivity in the area surrounding the glomeruli and epithelial tubules, (c) STZ-Nam-induced rats with reduced anti-Ins-positive cells in the islets of Langerhans, (d) STZ-Nam-induced rats with increased renal interferon-gamma positivity, (e) STZ-Nam-induced rats treated with TDN extract showing increased anti-Ins-positive cells, (f) STZ-Nam-induced rats treated with TDN extract showing reduced renal interferon-gamma positivity, (g) STZ-Nam-induced rats treated with quercetin with further increased anti-Ins-positive cells, and (h) STZ-Nam-induced rats treated with quercetin with further reduced renal interferon-gamma positivity. STZ refers to streptozotocin; S0 represents the control group; S1, the positive group; S2, the treatment group; and S3, the quercetin group. The cells were immunohistochemically stained, counterstained with hematoxylin, and viewed at 400× magnification.

In group S1, the islets of Langerhans exhibited severe inflammation and necrosis, whereas the glomeruli and tubules exhibited severe inflammation, necrosis, and hyaline accumulation (Figure 2). In addition, immunohistochemistry scores revealed a significant decrease in anti-Ins levels in group S1 compared with group S2 (p ≤ 0.05). Conversely, immunohistochemistry scores for IFN-γ indicated a significant increase in group S1 compared with group S2 (p ≤ 0.05) (Figure 3).

## DISCUSSION

The particle size results indicated that the TDNP emulsion exhibited significant stability. SEM analysis of particle size categorizes TDNP emulsions within the nanoparticle scale, with sizes ranging from 1 to 100 nm for ultra-smooth particles and 100 to 2500 nm for smooth particles. Particle size is a critical factor in nanoparticle systems because it determines *in vivo* distribution, toxicity, and targeting efficacy. In addition, particle size significantly affects drug loading, drug release, and the overall stability of nanoparticle systems [20, 21].

Previous studies by have demonstrated that STZ-nicotinamide induction increases BG, creatinine, urea, and γ-GT [17]. Hyperglycemia, creatinemia, and uremia are evident in DN rats owing to pancreatic islet cell destruction. However, in STZ-nicotinamide-induced diabetic neuropathic rats, some cells retain normal function and can still secrete insulin [22]. Conversely, in DN rats experiencing insulin resistance, normal insulin function is hindered despite elevated insulin secretion. The ZnO nanoparticle emulsion derived from *T. diversifolia* extract enhanced insulin action at the cellular level. For example, *T. diversifolia* leaf extract reduced glucose concentrations in rats with DN [23].

Quercetin, commonly found in green tea, has antidiabetic effects by increasing insulin levels and inhibiting sodium-dependent glucose transporter-1 formation. In STZ-Nam-induced rats, it prevents pancreatic islet cell activation. Normally, insulin acts as a hormone by signaling to cell receptors when glucose enters the bloodstream [24].

Consuming green tea significantly reduces fasting BG levels. Studies have shown that quercetin and quercetin-containing foods can improve hyperglycemia and dyslipidemia in type 2 diabetes patients. In addition, substantial quercetin intake leads to a notable decrease in plasma glucose levels by increasing glucose oxidation in skeletal muscles. Glucose oxidation provides energy, and its rate depends on the entry of glucose into cells. Therefore, increasing insulin levels may not always lower BG levels in DN and insulin resistance patients [25].

Our findings revealed that rats treated with TDNP and quercetin exhibited lower BG concentrations, creatinine, urea, γ-GT, and HDL-C compared with the S1 group. In particular, TDNP treatment significantly reduced BG concentration. The presence of flavonoids in *T. diversifolia* leaves exhibited antidiabetic effects by stimulating pancreatic β cells to release more insulin, thereby enhancing glucose utilization by skeletal muscles and inhibiting the action of α-glucosidase and amylase enzymes responsible for polysaccharide breakdown into monosaccharides. This reduction in glucose absorption contributes to the disruption of glucose homeostasis [26].

In individuals with diabetes mellitus, the body struggles to metabolize glucose, fatty acids, and amino acids effectively because of a lack of receptors. Consequently, a significant amount of glucose accumulates in the bloodstream, leading to elevated cholesterol levels and the triggering of various organ complications. Approximately half of the glucose consumed by patients with diabetes mellitus is converted into carbon dioxide, water, and fat in the adipose tissue, with less being stored as glycogen. In type 2 diabetes mellitus, an imbalance between glucose production and intake contributes to elevated BG levels. This prompts insulin to facilitate the entry of sugar into cells, particularly skeletal muscle cells. However, insulin resistance develops and is characterized by a surplus of insulin that cannot effectively perform its function. Consequently, glucose in the bloodstream cannot enter the cells, leading to a condition in which islet cells continue to produce insulin, resulting in hyperinsulinemia alongside hyperglycemia [27, 28].

TNF-alpha and IL-1β, key pro-inflammatory cytokines, play significant roles in the dysfunction of cells within the islets of Langerhans in type 2 diabetes. The presence of inflammation and necrosis strongly influences insulin secretion and triggers apoptosis in the pancreatic islets of Langerhans [29–31].

Previous studies have indicated that *T. diversifolia* leaves are rich in phenolic compounds and possess antioxidant properties measured in gallic acid equivalents. These compounds, along with alkaloids, tannins, flavonoids, saponins, and terpenoids, found in *T. diversifolia* leaves, stems, and roots, offer therapeutic benefits [32, 33]. Flavonoids, in particular, exert anti-diabetic effects by reducing oxidative stress and modulating glucose transporters. Enhanced GLUT-2 gene expression in beta-cells and facilitated GLUT-4 translocation, improving glucose metabolism. Saponins, on the other hand, regulate BG levels and prevent complications, thereby beneficial in managing diabetes, especially in individuals with chronic alcohol consumption. Moreover, saponin supplementation significantly reduces BG concentrations and improves plasma insulin levels [34, 35].

Quercetin, a substance commonly found in green tea, exerts its antidiabetic effects by increasing insulin concentration and inhibiting the formation of sodium-dependent glucose transporter-1. In rats treated with STz-Nam, quercetin prevents the activation of pancreatic islet cells. However, insulin acts as a hormone-signaling cell receptor under normal conditions when glucose molecules enter the bloodstream. Another study indicated that quercetin and quercetin-containing foods improved hyperglycemia and dyslipidemia in patients with type 2 diabetes mellitus. Moreover, the administration of substantial amounts of quercetin leads to a more pronounced reduction in plasma glucose levels, as quercetin induces a significant increase in glucose oxidation in skeletal muscles. In general, glucose oxidation provides energy, and its rate is contingent on the entry rate of glucose into cells. Consequently, elevated insulin concentrations may not necessarily reduce BG concentrations in patients with DN and insulin resistance [36, 37].

Our findings revealed significant differences in the concentrations of TNF-α, SOD, GPx, anti-ins, and IFN-γ between the treatment and control groups, as outlined in the tables. Specifically, TNF-α concentrations decreased notably in the treatment group compared with the positive control group, whereas SOD and GPx concentrations were significantly higher in the treatment groups. Interestingly, anti-ins levels were significantly increased in the treatment group (S2) compared with the control group (S1), whereas IFN-γ levels remained lower in the treatment group. The increase in anti-ins levels in the treatment group (S2) suggests a potential immunomodulatory effect of TDNP, possibly reflecting a compensatory mechanism to counteract immune-mediated β-cell destruction or enhance insulin signaling. This finding highlights the complex interplay between oxidative stress, inflammation, and immune regulation in the pathogenesis of diabetes. The elevated SOD and GPx concentrations in the treatment group confirmed the anti-oxidant properties of TDNP. These enzymes play critical roles in neutralizing reactive oxygen species, thereby reducing oxidative stress - a key contributor to insulin resistance and β-cell dysfunction. The antioxidant components of TDNP leaves are likely responsible for this protective effect.

Furthermore, the reduction in TNF-α concentrations in the treatment group was associated with an improved inflammatory status. TNF-α is a pro-inflammatory cytokine that impairs insulin signaling and intensifies insulin resistance. The observed decrease suggests that TDNP mitigates oxidative stress and suppresses inflammatory pathways. Although interferon- levels were lower in the treatment group, this reduction could indicate a dampened Th1-mediated immune response, further supporting the immunomodulatory role of TDNP. Together with the increased anti-ins levels, these findings suggest that TDNP may help restore a balanced immune environment, which is conducive to better insulin action.

In summary, the results demonstrate that TDNP treatment improves oxidative stress markers (SOD and GPx), reduces inflammation (TNF-α), and modulates immune responses (anti-ins and IFN-γ). These effects enhance insulin action and protect against β-cell damage in rats with insulin resistance induced by STZ-nicotinamide. These findings support the therapeutic potential of TDNP in managing diabetes and its complications.

Furthermore, our results demonstrated that TDNP treatment decreased TNF- concentrations compared with the positive control group. Previous studies [38, 39] have highlighted *T. diversifolia*’s phenolic components and antioxidant properties, which are known to reduce cholesterol and low-density lipoprotein concentrations. In addition, TDNP has been shown to improve the lipid profile, reduce body fat, and lower total cholesterol concentrations in patients with DN. These studies have also established a positive correlation between TNF-alpha concentrations, triglyceride levels, HDL, BG, and hypertension in individuals with DN.

Our study used a monoclonal antibody against IFN-γ to detect its presence in renal tubule epithelial and mesangial cells. The concentrations of IFN-γ showed a regulatory effect on BG levels, with reductions observed in both the treatment and quercetin groups. The observed reduction in cellular glucose uptake may be linked to the proliferation of IFN-γ-positive cells. Compared with the treatment group (S2), the positive control group (S1) exhibited a higher distribution of renal IFN-γ-positive cells in affected tubule epithelial and mesangial cells. These findings suggest variations in the distribution of IFN-γ-positive cells within the interstitial tubules in the TDNP-treated groups. Furthermore, increased numbers of renal IFN-γ-positive cells were consistently observed in STZ-Nam-induced rats in the S2 and S3 groups, which were correlated with changes in TNF-α concentrations.

Histopathological examination of the pancreas revealed severe tissue damage characterized by cellular inflammation and necrosis in the islet area, resulting in loss of the Langerhans islet structure. Vacuole formation and significant loss of β cells were observed. Immunostaining showed decreased anti-ins-positive cells in group S1, indicating activation of inflammatory signaling pathways in STZ-Nam-induced rats. TDNP treatment significantly restored BG concentrations to normal levels, surpassing the effects of quercetin. In addition, TDNP extract significantly increased the levels of SOD and GPx compared with the control group (S0 and S1). Previous studies by have demonstrated the efficacy of nanoparticles derived from natural substances in improving BG and insulin levels in diabetic-induced rat models [40, 41].

The observed effects of *T. diversifolia* extract in a ZnO nanoparticle emulsion in DN rats are promising and warrant further consideration. Our study demonstrated that TDNP effectively reduced TNF-α levels, indicating its potential to mitigate inflammation in DN. In addition, TDNP treatment increased the concentrations of SOD and GPx, suggesting an enhancement in antioxidant defense mechanisms. These findings agree with previous studies by highlighting the antioxidant properties of *T. diversifolia* [42, 43]. Furthermore, anti-ins expression was enhanced in the TDNP-treated group, indicating a potential role in preserving pancreatic function and insulin sensitivity. This is particularly noteworthy because preserving pancreatic function is crucial for managing BG levels in individuals with diabetes. Moreover, the observed reduction in IFN-γ expression suggests a regulatory effect on BG concentrations, potentially contributing to improved glucose homeostasis. Overall, our study underscores the potential of TDNP in mitigating inflammation, enhancing antioxidant defense, and preserving kidney function in patients with DN.

These findings offer valuable insights into the therapeutic effects of TDNP and underscore the need for further investigation to elucidate its underlying mechanisms of action and its potential as a treatment for DN. However, our study had limitations, including the need to provide more details about the origin and collection methods of the plant extract, the necessity for more frequent monitoring of glycemia during diabetes induction, and the need for a more detailed morphologic description of kidney lesions.

## CONCLUSION

This study demonstrated that feeding TDNP emulsion effectively ameliorated key biochemical and histopathological markers of DN in STZ-induced diabetic rats. TDNP treatment significantly reduced blood glucose, creatinine, urea, and TNF-α concentrations while increasing antioxidant markers such as SOD and GPx. Furthermore, it enhanced pancreatic anti-insulin expression and reduced kidney interferon-gamma (IFN-γ) levels, indicating improved insulin regulation and reduced inflammation. Histopathological findings corroborated these results, revealing decreased necrosis and inflammation in both pancreatic and renal tissues.

The strengths of this study include the use of a well-established DN model that closely mimics human type 2 diabetes and the comprehensive evaluation of oxidative stress, inflammatory markers, and histological changes. In addition, the novel integration of *T. diversifolia* and zinc oxide nanoparticles highlights a promising therapeutic approach with potential synergistic effects.

However, the study has several limitations. The sample size was limited, and the findings may not fully translate to humans without further validation. While the study provided detailed biochemical and histological assessments, long-term effects and potential toxicity of TDNP were not investigated. Moreover, the precise molecular mechanisms underlying TDNP’s therapeutic effects remain unclear.

Future studies should focus on elucidating the molecular pathways modulated by TDNP, investigating its long-term safety and efficacy, and evaluating its therapeutic potential in clinical settings. Exploring its effects in combination with standard DN treatments could provide additional insights. Expanding research to include various doses and extended treatment durations may also offer a deeper understanding of its pharmacodynamics and optimal therapeutic window.

TDNP shows significant promise as a natural, nanoparticle-enhanced therapeutic agent for managing DN by mitigating oxidative stress, reducing inflammation, and improving insulin regulation. Further research is warranted to translate these findings into clinical applications and establish its role in comprehensive diabetes management.

## AUTHORS’ CONTRIBUTIONS

ISH and RS: Conceptualized the study, conducted blood sampling, analyzed data, and drafted the manuscript. RS and LM: Conducted the laboratory study and study design. STM and LM: Interpreted the results, analyzed data, and drafted the manuscript. All authors have read and approved the final manuscript.
